# Social and Poor-Law Problems

**Published:** 1907-08-31

**Authors:** 


### SOCIAL AND POOR-LAW PROBLEMS.

### FREE FOOD AND LAZINESS.

When the last annual census of London's homeless poor was taken it revealed that about 2,400 of the inhabitants of the metropolis had neither shelter nor the means to procure it. The proportion to the total inhabitants of London—about 1 in 2,000—remains much the same as in previous takings of numbers, which, though at best only approximate, are fairly reliable. It is a comfort, such small comfort as we can find in the matter, that among the homeless there were only four children, the remaining numbers being made up of 1,998 men and 402 women. A considerable number of the homeless were receiving food at the depots of the Church and Salvation Armies, and other missions are engaged in feeding the hungry without any inquiry as to the cause of the hunger. At a mission hall in Shoreditch about 600 men are fed daily at a very low cost, a substantial meal being obtainable at 2d. or 3d., and in the same place free food is distributed to 350 men each Sunday and 900 men each Thursday. On the Embankment any number up to 1,500 are supplied with soup and bread without charge. Sir Shirley Murphy, who presents the report of the census, says that, in his opinion, it is possible for a man to live on a penny a day in London, if he is indifferent to the matter of shelter, and knows how to take advantage of the supplies of free and cheap food available. The constancy of the proportion of the homeless to the total population raises the question as to whether all these charitable agencies are really doing much permanent good. Whether trade is bad or good the number of these wanderers does not seem to alter. They sleep anywhere, and pick up their food for as nearly nothing as circumstances allow. One would gladly assume that they are men out of work for good and honourable reasons, but this constancy of proportion seems to cast doubt upon that. And for the genuinely unfortunate there are workhouses, and for the chance wanderer there are casual wards. For those who can scrape up 6d. a night there are Rowton Houses and common lodging-houses. In face of all this, it seems doubtful if there is much need for extra agencies. The religious bodies indeed hope to win men's souls through gifts to their bodies, but where there is no test it seems doubtful if they achieve their aim. And there is always the risk of breeding a race of loafers, able to live without working, skilled in the location of free food depots, and skilled in nothing else. To encourage these is to encourage the submerged tenth to swamp the rest of the nation.

### WORKHOUSE MEDICAL OFFICERS AND FEES FOR OPERATIONS.

A proposal has been laid before the Local Government Board that the medical officer of a workhouse should receive special fees for performing operations. This, however, the Board declines to sanction, as the salary of the medical officer ought to be sufficient to remunerate properly all his personal services. If, however, a medical officer requires to call in assistance, either in the administration of anæsthetics or otherwise, the Board is prepared to consider any application for the payment of gratuities to the gentleman who gave the necessary assistance. As a matter of fact, many medical officers of workhouses, like many general practitioners, prefer not to undertake any but simple operations. The present regulations of the Local Government Board permit of their calling in a surgeon for an operation of any magnitude, and would sanction a reasonable payment to the latter, and there seems to be no sufficient reason for making any change in the present methods, which answer well for the patients, and relieve the medical officer of a responsibility which might often be very serious.

